# Are empty methadone bottles empty? An analytic study

**DOI:** 10.1186/1477-7517-11-20

**Published:** 2014-07-02

**Authors:** Gaël Dupuy, Lia Cavalcanti, Emmanuel Bourgogne, Clara Brichant-Petitjean, Léon Gomberoff, Vanessa Bloch, Frank Bellivier, Jean-Pierre Lépine, Olivier Laprévote, Florence Vorspan

**Affiliations:** 1 Assistance Publique-Hôpitaux de Paris (AP-HP), Service de Psychiatrie, Hôpital Fernand-Widal, 200, rue du Faubourg St-Denis, Paris 75010, France; 2 Unité Variabilité de Réponse aux Psychotropes, Inserm U1144, Universités Paris-Descartes et Paris Diderot, PRES Sorbonne Paris Cité, 4, Avenue de l’Observatoire, Paris 75006, France; 3 Aurore, Association EGO, 6 rue de Clignancourt, Paris 75018, France; 4 AP-HP, Hôpital Lariboisière, Service de Toxicologie Biologique et Pharmacologie, 2 rue Ambroise Paré, Paris 75010, France; 5 Laboratoire de Chimie, Toxicologie Analytique et Cellulaire, CNRS UMR 8638 COMETE, Faculté de Pharmacie, Université Paris-Descartes, PRES Sorbonne Paris Cité, 4, Avenue de l’Observatoire, Paris 75006, France

**Keywords:** Methadone, Empty bottles, High-performance liquid chromatography

## Abstract

**Background:**

Methadone maintenance treatment is the most widely prescribed treatment for opiate dependence with proven benefits for patients. In naïve users or in case of recreational misuse, methadone can be a source of potentially lethal intoxications, resulting in fatal overdoses. A few cases of infantile intoxications have been described in the literature, some of which resulted in death. Nowadays, more than 50,000 bottles are used every day in France, most of which are thrown away in the bin. Relatives at home, especially children, can have access to these empty bottles. This study aims to determine whether the residual quantity of methadone in the bottles is associated with a risk of intoxication for someone who has a low tolerance to opiates, such as a child.

**Methods:**

The methadone dosage left in a sample of 175 bottles recapped after use by the patients taking their maintenance treatment in an addiction treatment program centre was analysed during a 2-week period in March 2013.

**Results:**

The mean residual quantity of methadone left in each bottle after use is 1.9 ± 1.8 mg and 3.3 ± 2.4 mg in the sample of 60 mg bottles.

**Conclusions:**

There is a potential danger of accidental overdose with empty bottles of methadone syrup, especially for children. To take into account this hazard, several harm reduction strategies can be proposed, such as favouring the taking of the treatment within the delivery centres rather than the ‘take home’ doses, asking methadone users to bring back their used bottles, and raising patients’ awareness of the intoxication risks and the necessary everyday precautions. For stable patients with take home methadone, the use of capsules could be considered.

## Background

Methadone maintenance treatment is the most widely prescribed treatment for opiate dependence with proven benefits for patients. In France, 45,442 patients are treated by methadone syrup in 2012 with a mean dose of 60 mg/day (60% of methadone prescriptions) [1]. This mean dose represents between one and three bottles used every day by each patient. The frequency of opiate substitution therapy in adults has tripled in the past decade. Nowadays, more than 50,000 bottles are used every day in France, most of which are thrown away in the bin. Nevertheless, some residents of areas where drug dealing is high as well as social workers have raised concern about empty bottles of methadone left behind in the streets, in the same way with that of syringes and pill boxes. Moreover, relatives at home, especially children, can have access to these empty bottles, either left behind or found in the bin.

In naïve users or in case of misuse, methadone can be a source of potentially lethal intoxications, resulting in fatal overdoses [2]. A few cases of infantile intoxications have been described in the literature, some of which resulted in death [3-9]. In all those cases, methadone was not prescribed for infants; the children got intoxicated by taking methadone that had been carelessly stored by their parents or by parental administration of the drug [5]. The following reasons may account for the delay in seeking help: methadone users may not realise the danger of the drug to children and may not know that there is an antidote. Secondly, parents may fear professional accusations of poor parenting. Therefore, the issue concerning the potential harmful effect of ‘empty or half empty’ bottles of methadone after use remains.

A report from the French Toxicovigilance Coordination Committee [10] carried out on the paediatric intoxications by methadone capsules and syrup between 2008 and 2012 in France reported 33 intoxications by methadone syrup, one of which was lethal, and 20 intoxications by methadone capsules (a total of 53 intoxications). The average age of the intoxicated children was 2 years old, and the average dose presumably swallowed was 15 mg with syrup forms. In most cases, the treatment was on a table, within the child's reach (bottles already opened or opened by the child). It should be noted that there was a case of intoxication in a public park, and four cases occurred in adolescents who did take methadone on purpose, to get ‘high’. It is not precisely mentioned if those subjects were naïve or tolerant. In terms of toxicity data, the toxic and potentially lethal methadone dose is 1 mg/kg for a naïve subject or for someone without acquired tolerance. In human subjects, the lowest published toxic dose (TDLo) ranges from 0.76 to 2 mg/kg, and the lowest published lethal dose (LDLo) is 1.3 mg/kg [11]. We therefore consider that a dosage of 40 mg for an adult and of 10 mg for a child whose weight is 10 kg can be potentially lethal [12].

Is the residual quantity of methadone in the empty bottles insignificant or is there a risk of intoxication for someone who does not tolerate opiates, such as a child? In order to answer this question, we collected from bins a sample of bottles recapped after use by the patients taking their maintenance treatment in our addiction treatment program centre (CSAPA Espace Murger) located in the Fernand-Widal Hospital, Paris. The analysis of the methadone dosage left in these bottles was carried out in the toxicology laboratory of the Lariboisière Hospital in Paris.

## Methods

The CSAPA Espace Murger allows patients to take their daily dose of methadone in a special delivery room with a nurse and can, in this way, offer a safe monitoring of the treatment delivery. They can choose to rinse their bottles with water and drink again or not to; they then throw their empty bottles in a special medical waste disposal container ready for incineration. In March 2013, our staff asked all patients to make sure they recapped their bottles (in order to collect all the residual syrup) each time they came to take their treatment, before they threw them away in the bin. Each time the bin was full, our staff emptied it and collected all the bottles inside, except for those who had not been recapped. At the end of a 2-week period, 175 bottles were collected.

### Biological analysis

The concentrations of methadone were determined in all syrup samples. The syrup samples (50 μL) were diluted 2,000-fold to 5,000-fold with deionized water prior to analysis. The diluted samples were mixed with a solution of the deuterated methadone (methadone-d3), used as an internal standard. The extracts were analysed using an on-line turbulent flow SPE LC-MS/MS. An Aria Transcend TLX-1 System (ThermoFisher Scientific, San Jose, CA, USA) consisting of two Accela 600 high-pressure quaternary pumps (one loading pump for the TurboFlow system and one eluting pump for the analytical column system), a valve interface module and a CTC PAL Autosampler (LEAP Technologies, Inc., Carrboro, NC, USA) was used. For loading and eluting pumps, the system eluents used were (A) 0.1% formic acid in water, (B) ammonium hydrogen carbonate pH 9 in water and (C) 0.1% formic acid in methanol. The entire experiment was controlled by Aria operating software 1.1.1. In the first step, 30 μL supernatant from the sample preparation was injected and loaded onto a C18 XL TurboFlow column (50 mm × 0.5 mm internal diameter (i.d.), ThermoFischer Scientific) under turbulent flow (100% B, 1.5 mL/min, 30 s). In the second step, retained analytes were flushed from the TurboFlow column using an elution solvent (A-C, 97/3, *v*/*v*) stored in a holding loop and focused through a Tee piece on to a Hypersil Gold C18 analytical column (3-μm particle size, 100 mm × 2.1 mm i.d.; ThermoFischer Scientific). In the third and fourth steps, during gradient elution (A-C, 97/3 to 10/90 *v*/*v*) from the analytical column, the TurboFlow column was washed with eluent C (100%). In the fifth step, the loop was refilled with the elution solvent (A-C, 97/3 *v*/*v*) while the chromatographic separation was proceeding on the analytical column. In the sixth step, the loading and eluted systems were re-equilibrated at initial conditions. Mass spectrometry detection was achieved using a Quantum Ultra mass spectrometer (ThermoFischer) coupled to the Aria TLX-1 system. The mass spectrometer was set to admit the protonated molecular ions (M + H)^+^ in the first quadrupole. These precursor ions were fragmented by collision-induced dissociation with argon in the second quadrupole. Product ions specific of each compound were monitored in the third quadrupole. Selected reaction monitoring transition were *m/z* 310.1 → 105 (quantification ion) and 310.1 → 265.2 (qualification ion) for methadone and *m/z* 313.1 → 268.2 for methadone-d3 (deuterated internal standard). The lower limit of quantification was 50 ng/mL. The analytical runtime was 11 min, with methadone eluting at 7.5 min. The response was linear over the concentration ranges of 50 to 1,000 ng/mL. Inaccuracy was ≤2.9%, and the relative standard deviation was ≤11.3% across the quantification range. This assay allowed the estimation of methadone in these 175 commercial syrups. For this entire study, over 30 quality control samples were run. Accuracy and precision were 106.8% and 8.40%, respectively, which fulfilled the criteria for bioanalytical validation [13,14].

## Results

**Figure 1:**
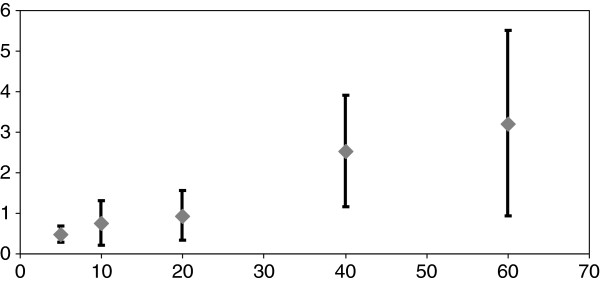
**Distribution of the residual quantity of methadone for each methadone dosage (5-, 10-, 20-, 40- and 60-mg bottles).** Mean ± SD (ANOVA: *F* (4 *df*) = 21; *p* < 0.001).

The results were obtained from the analysis of the selected 175 bottles. There were 2 bottles of 5 mg (1.1%), 36 of 10 mg (20.6%), 43 of 20 mg (24.6%), 39 of 40 mg (22.3%) and 55 of 60 mg (31.4%). The mean residual volume was 0.73 mL/bottle (0–1.9), and the mean residual quantity of methadone was 1.9 ± 1.8 mg/bottle (0–7.2). Figure 1 shows the distribution of the residual quantity for each methadone initial dosage. The quantity was different according to the initial quantity of the bottle (0.5 ± 0.2 vs 0.7 ± 0.5 vs 0.9 ± 0.6 vs 2.5 ± 1.4 vs 3.2 ± 2.4 for 5, 10, 20, 40 and 60 mg respectively; ANOVA: *F*(4 *df*) = 21; *p* < 0.001).

**Figure 2:**
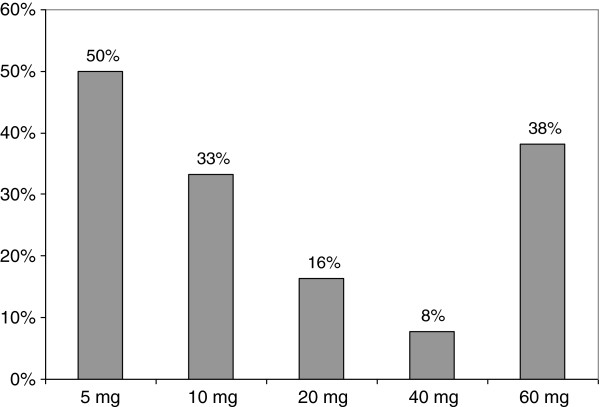
Percentage of rinsed bottles for each initial methadone dosage (5, 10, 20, 40 and 60 mg).

One of the conclusions that can be drawn from these results is that since 10 mg of methadone for a child (who weighs 10 kg) can be potentially lethal [11], then a child would have to drink three bottles of ‘empty’ 60-mg bottles (mean residual quantity = 3.2 ± 2.3 mg) to have a fatal overdose. Moreover, only 44 bottles analysed (25%) had low residual volumes and low methadone concentration. We hypothesize that those bottles are the one that have been rinsed by patients with water in order to drink all the residual syrup. Figure 2 shows the percentage of rinsed bottles for each methadone dosage.

## Discussion

We performed an analytic study assessing the residual methadone dosage left in empty bottles by treated patients. It shows that the mean residual quantity of methadone left in each bottle is 1.9 ± 1.8 mg, but 3.2 ± 2.3 mg in the sample of 60-mg bottles. Therefore, in a context of increase of methadone prescription in France and of an already observed increase of accidental and non-accidental ingestion of methadone in childhood in other countries [15], the potential risk of accidental overdose in case of ingestion of such bottles by a child cannot be ruled out. Although, no intoxication with empty methadone bottles was ever reported in French pharmacovigilance databases, we suggest that this hazard needs to be taken into account, and there are several easy ways to implement harm reduction policies. First, proper information should be given to the patients on the dangers of methadone intoxication for non-tolerant patients, especially that there has been an increase in methadone prescription in adults over the past decade. Patients should be informed that accidental overdose by a child is a medical emergency and can result in death. The risk management plan issued from the French National Agency for the Safety of Medicines and Health Products (ANSM) includes information campaigns (posters and leaflets) designed to warn and advise users about the paediatric intoxication risk of methadone capsules. These warnings should also include the risk related to the use of empty methadone bottles left to the reach of children. Secondly, it is also important for health care professionals to provide thorough information and guidance to methadone users on the safe storage and the necessary precautions that must be taken with empty bottles, i.e. ‘take-home’ methadone should not be thrown away on the streets but should be kept in a safe place, out of sight and reach of children, and the bottles should be rinsed out and securely closed before being thrown away in the bin. Furthermore, methadone users could be encouraged to bring back their used bottles, maybe with a voucher for returnable bottles. Finally, for stable patients who received take-home methadone, this result provides an argument to propose the systematic prescription of methadone capsules since there can be no residual dosage left. It should be noted that this intoxication with empty bottles problem does not exist with the capsules form. Moreover, according to the French Toxicovigilance Coordination Committee's report [10], 8 out of 33 security bottle caps were opened by children (one of whom was under 3 years old), which has never been the case with the *child*-*proof* capsule packages. Furthermore, this report did not describe any differences between the two forms of methadone in terms of risk of paediatric accidents after 4 years of study. As far as methadone clients are concerned, all of those interviewed in a study in France on the acceptability of capsule formulation recognized the contribution of this new formulation on use, side effects and transport [16].

## Conclusion

To conclude, our analytic study did not rule out a potential danger of accidental overdose with empty bottles of methadone syrup, especially for children. The risk reduction strategies could favour the taking of the treatment within the delivery centres rather than the take home doses for unstable patients, to raise patients' awareness of the intoxication risks and the necessary everyday precautions. Empty methadone bottles should be cleared off the streets in the surroundings of illegal drug-dealing market places. For patients who are stable and receive take-home methadone, the risk reduction strategies could include the use of returnable bottles or to consider the use of capsules.

## Competing interests

The authors declare that they have no competing interests.

## Authors’ contributions

GD and CBP wrote the first draft of the manuscript. LC, LG, JPL and FB designed the study. FV, GD, VB, EB and OL performed the analytical study. All authors provided critical revision of the manuscript and approved the final version.
